# Epigenome-Wide Analysis of DNA Methylation in Parkinson’s Disease Cortex

**DOI:** 10.3390/life12040502

**Published:** 2022-03-29

**Authors:** Oliver Kaut, Ina Schmitt, Fabian Stahl, Holger Fröhlich, Per Hoffmann, Frank J. Gonzalez, Ullrich Wüllner

**Affiliations:** 1 Department of Neurology, University of Bonn, 53105 Bonn, Germany; ina.schmitt@ukbonn.de (I.S.); ullrich.wuellner@ukbonn.de (U.W.); 2 DZNE (Deutsches Zentrum für Neurodegenerative Erkrankungen), 53105 Bonn, Germany; fabian.stahl@dzne.de; 3 Algorithmic Bioinformatics for B-IT, University of Bonn, 53113 Bonn, Germany; frohlich@bit.uni-bonn.de; 4 Life & Brain Center, Institute for Human Genetics, Department of Genomics, University of Bonn, 53113 Bonn, Germany; per.hoffmann@unibas.ch; 5 Human Genetics Research Group, Department of Biomedicine, University of Basel, 4001 Basel, Switzerland; 6 Laboratory of Metabolism, National Cancer Institute, Bethesda, MD 20892, USA; fjgonz@helix.nih.gov

**Keywords:** Parkinson’s disease, cortex, epigenetics, DNA methylation

## Abstract

Background: Epigenetic factors including DNA methylation contribute to specific patterns of gene expression. Gene–environment interactions can change the methylation status in the brain, and accumulation of these epigenetic changes over a lifespan may be co-responsible for a neurodegenerative disease like Parkinson’s disease, which that is characterised by a late onset in life. Aims: To determine epigenetic modifications in the brains of Parkinson’s disease patients. Patients and Methods: DNA methylation patterns were compared in the cortex tissue of 14 male PD patients and 10 male healthy individuals using the Illumina Methylation 450 K chip. Subsequently, DNA methylation of candidate genes was evaluated using bisulphite pyrosequencing, and DNA methylation of cytochrome P450 2E1 (*CYP2E1*) was characterized in DNA from blood mononuclear cells (259 PD patients and 182 healthy controls) and skin fibroblasts (10 PD patients and 5 healthy controls). Protein levels of *CYP2E1* were analysed using Western blot in human cortex and *knock-out* mice brain samples. Results: We found 35 hypomethylated and 22 hypermethylated genes with a methylation M-value difference >0.5. Decreased methylation of cytochrome P450 2E1 (*CYP2E1*) was associated with increased protein levels in PD brains, but in peripheral tissues, i.e., in blood cells and skin fibroblasts, DNA methylation of *CYP2E1* was unchanged. In *CYP2E1 knock-out* mice brain alpha-synuclein (*SNCA*) protein levels were down-regulated compared to wild-type mice, whereas treatment with trichloroethylene (TCE) up-regulated *CYP2E1* protein in a dose-dependent manner in cultured cells. We further identified an interconnected group of genes associated with oxidative stress, such as Methionine sulfoxide reductase A (*MSRA*) and tumour protein 73 (*TP73*) in the brain, which again were not paralleled in other tissues and appeared to indicate brain-specific changes. Conclusions: Our study revealed surprisingly few dysmethylated genes in a brain region less affected in PD. We confirmed hypomethylation of *CYP2E1*.

## 1. Introduction

Epigenetic factors, particularly DNA methylation, modify gene expression and have been shown to undergo dynamic changes throughout life and in differentiated neurons of the human brain [1,2]. Methylation changes that occur in response to early developmental challenges and environmental exposures are carried forward in the cells’ DNA and may be partially passed on to offspring [3]. Persistent epigenetic differences in men have been observed after prenatal exposure to famine and have been associated with several pathologic conditions, including neurodevelopmental disorders [4]. Thus, altered DNA methylation and consecutively altered gene expression patterns likely contribute to the individual susceptibility towards neurodegenerative diseases in general and PD in particular [5,6]. However, the DNA methylation levels in the brain when comparing healthy individuals and PD patients showed no differences in several relevant genes, like UCHL1 (ubiquitin carboxyl-terminal hydrolase 1 gene), MAPT (microtubule associated protein tau promoter) and PRKN (parkin) promoter [7,8].

Several huge GWAS in Parkinson’s disease found the SNCA gene as a highly significant genetic risk factor (reviewed in [9]). DNA methylation patterns in PD brains of the CpG islands in intron 1 of SCNA have been investigated, but showed contradictory results [10,11,12]. This may be due to cell-type specific differential methylation in different brain cell populations. Recently, Gu et al. demonstrated DNA hypomethylation in sorted neuronal nuclei from the PD frontal cortex in comparison to controls, but no difference in sorted glia nuclei.

We here extended previous studies to a larger sample of cortical tissues from male PD patients and healthy controls, avoiding a confounding effect of imprinting by sex, using Illumina 450 K methylation bead arrays. Consistent with our preliminary data, we identified cytochrome P450 2E1 (CYP2E1) among the most intensely and repetitively hypomethylated genes. Independent techniques, i.e., bisulphite pyrosequencing (BPS) at single-base resolution, confirmed *CYP2E1* hypomethylated, specifically in the cortex DNA from PD patients, associated with increased protein levels. In Cyp2e1-null mice, we observed decreased alpha-synuclein protein, suggesting that CYP2E1 in wild-type mice (and men) might contribute to increased alpha-synuclein expression.

## 2. Methods

### 2.1. Study Population

DNA was extracted from 24 cortex samples from 14 male PD patients (mean age ± SD: 77.7 ± 5.9 years) and 10 male neurologically healthy individuals (mean age ± SD: 76.0 ± 8.75 years) provided by the Brain Bank Munich and by Professor Schulz-Schaeffer. DNA extraction and bisulphite conversion were performed as described previously [5].

### 2.2. Epigenome-Wide Methylation Analysis

Four hundred nanograms of bisulphite-treated DNA were analysed using the Infinium Human Methylation 450 K bead array according to the manufacturer’s instructions. The Ethics Committee of the Medical Faculty of the University of Bonn approved the study (No. 51/00, 6 July 2000).

### 2.3. Statistical Analysis of Normalised Methylation Data

Illumina GenomeStudio^®^ software (version 2011.1) was used for the extraction of DNA methylation signals as raw signals without background normalization; methylation data were further processed via the Bioconductor lumi package using shift-scale colour bias adjustment and quantile normalization as further pre-processing steps implemented in the lumi package [13]. A detection *p*-value cut-off of 0.00001 was used to filter out signals below background, which left 320.898 CpG for testing.

### 2.4. Pyrosequencing of Cortex Samples

A PyroMark Q24 System (Qiagen) was used for DNA methylation analysis (Primer: *CYP2E1* PCR: PF1_GGGGTTGTTTTTGAGTAGGAGT and PR1_TCAATAAATCTCTTCCCCCTT C, Pyrosequencing: PS1_TTTTTATTTATGTTGAGG; C21orf55:PF1_TTATGAGGATGAG ATGATTTATTTG and PR1_Bio_CCCTAACTCCCTACTTCAATTAC; PS1_GAGATGAT TTATTTGTTGT; TP73: PF1_GGTTTAATAGGGAGTGGTAGTTATTTT and PR1_Bio_A CCCACCCTAACACTAACCA, PS1_AGTGGTAGTTATTTTAAAGG). Statistical analysis was performed using SPSS Statistical software 20.0 (SPSS Inc., Chicago, IL, USA). Values are indicated as the mean ± SD, and comparisons between groups were performed using the Mann–Whitney U test. The level of significance was set at *p* < 0.05 and <0.01, respectively.

### 2.5. Pyrosequencing in the Cortex and Peripheral Blood with CYP2E1 Primer 2

The pyrosequencing primer pairs were designed by O. Jiménez-Garza [14].

### 2.6. Pyrosequencing in Human Peripheral Blood Mononuclear Cells (PBMCs)

Identical primers to those applied for pyrosequencing of cortex samples were used for blood samples. DNA from 259 PD patients (aged 63.0 ± 10.13 years; 167 male; 92 female) and 182 healthy controls (aged 58 ± 13.85 years; 123 male; 59 female) was analysed.

### 2.7. Pyrosequencing in Human Peripheral Blood Mononuclear Cells (PBMCs) Exposed to l-DOPA

The methylation of DNA derived from the pbms of 4 male de novo PD patients without prior l-DOPA medication (aged 63.2 ± 9.49) was analysed using pyrosequencing before and after pbms were exposed to 100 µM and 200 µM l-DOPA for 48 h.

In addition, we compared the methylation state of *CYP2E1* in DNA from the blood of PD patients without prior l-DOPA intake (*n* = 11; mean age: 64.4 years), low l-DOPA dosage (<300 mg/d; *n* = 8; mean age: 70.3 years) and high l-DOPA dosage (>1000 mg/d; *n* = 16; mean age: 66.5 years).

### 2.8. Pyrosequencing in Skin Fibroblasts

DNA from cell-cultured skin fibroblasts from 5 healthy probands (3 males; mean age ± SD: 55.3 ± 22.8 years, age and sex missing in 2 samples) and 12 PD-affected patients (10 males, 2 females; mean age ± SD: 67.7 ± 14.84 years) was used for pyrosequencing.

### 2.9. Western Blot Analysis

Western blot analysis was performed using 50 µg of protein per lane and mouse monoclonal anti-*β*-actin (clone AC-15; A5441; Sigma, St. Louis, MO, USA) and rabbit polyclonal anti-*CYP2E1* (ab53945, abcam, Cambridge, MA, USA) antibodies. Densitometric quantification and normalization to the *β*-actin levels were performed using LabImage 1D L340 (Intas Science Imaging; Göttingen, Germany).

### 2.10. CYP2E1 Knock-Out Mice

*CYP2E1*-null mice were described previously [15]. We used brains of five CYP2E1-null mice and four brains of wild-type mice as the control.

## 3. Results

### 3.1. Epigenome-Wide DNA Methylation Analysis in Brain

The epigenome-wide methylation analysis revealed 57 differentially methylated genes, but without reaching the level of significance after *p*-value adjustment.

**Figure 1:**
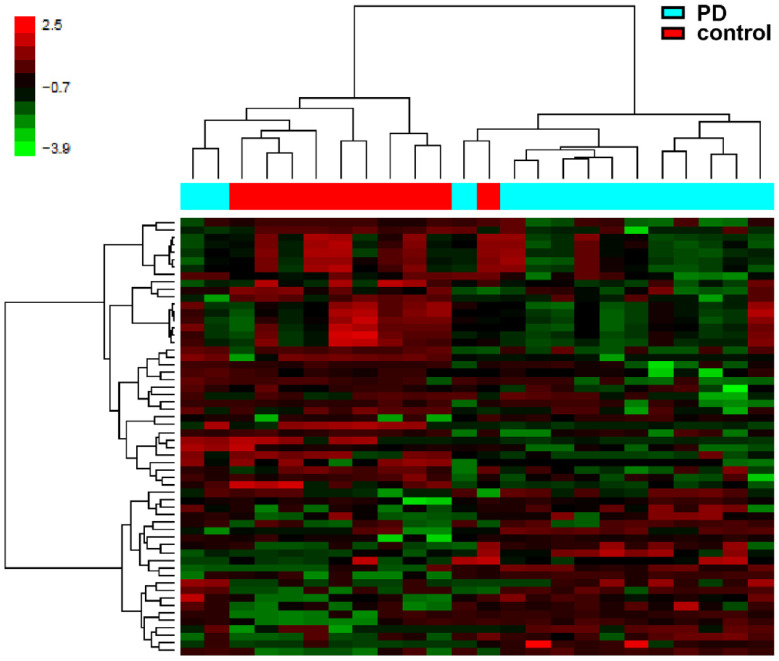
Heat map of DNA methylation in cortical samples. The heat map displays highly methylated loci in red and sparsely methylated loci in green. Hierarchical clusterings (average linkage) of the samples after normalization is based on all CpGs. Hierarchical clustering analysis reveals a clear separation of the control vs. PD cases.

We found 35 hypomethylated and 22 hypermethylated in PD, with a methylation difference >0.5 of the M-value (Table 1). The corresponding segregation of PD patients and controls is visualized in a heat map plot and hierarchical clustering analysis (Figure 1). Differentially methylated CpGs were mainly located in gene bodies (66%), and only a few were found upstream of the transcription start site (TSS 1500; 15%), in the 5′UTR (14%) and 3′UTR (5%).

Two genes were identified with multiple differentially methylated CpGs: *CYP2E1* (5 CpGs hypomethylated in PD) and *C21ORF56* (6 CpGs hypomethylated in PD). Large differences regarding the degree of methylation were found again in *CYP2E1*, but also in methionine sulfoxide reductase A (*MSRA*, hypomethylated in PD) and cadherin 13 (*CDH13*, hypermethylated in PD) (Table 1).

### 3.2. Pyrosequencing of Candidate Genes in Brain

**Figure 2:**
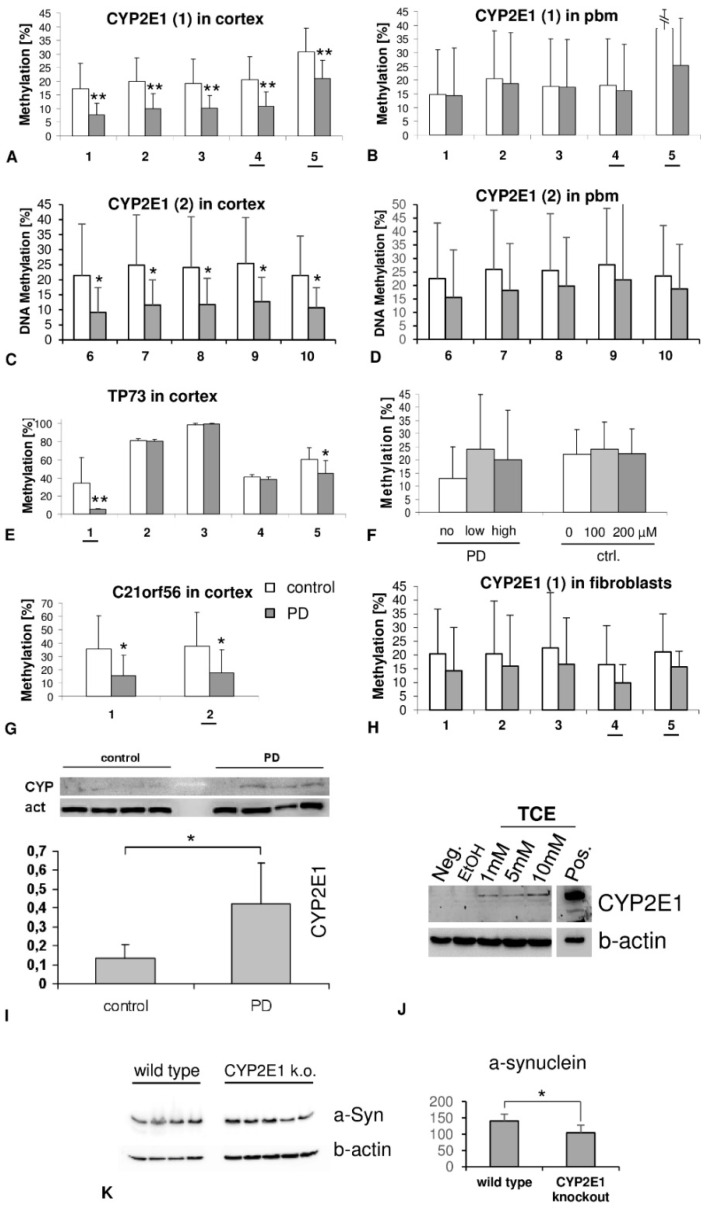
Pyrosequencing and western blots of candidate genes A–H. Evaluation of DNA methylation using pyrosequencing. We confirmed the methylation status of *CYP2E1* (**A**), *TP73* (**E**) and *C21orf56* (**G**) in cortical samples. In the pbms, no difference in *CYP2E1* methylation was observed (**B**). A different region (primer pairs of [14] of the *CYP2E1* gene was also hypomethylated in the brain (**C**) but not in blood monocytes (**D**). L-DOPA treatment had no obvious effect on *CYP2E1* DNA methylation in PD patient pbms (Pearson coefficient 0.019; *p* = 0.92) (**F**). In skin fibroblasts, the differential methylation was unchanged when comparing cases and controls (**H**). The underlined CpGs correspond to the CpG site annotated on the methylation microarray. Using western blot analysis (**I**), significantly increased expression levels of *CYP2E1* in Parkinson-diseased brains (*n* = 4) were found compared with the control (*n* = 4) (control 0.13 ± 0.07; PD 0.42 ± 0.21; *p* = 0.042). Equal loading was controlled by the immunodetection of beta-actin (b-actin). Results were presented as the mean ± SD of the control and PD groups (* *p* < 0.05; ** *p* < 0.01). In cultured Sk-H-SH cells, exposure to trichloroethylene (TCE) induced *CYP2E1* up-regulation in a dose dependent manner (**J**). In the brain samples of *CYP2E1* null-mice, a-synuclein protein was significantly decreased (**K**).

To validate the array data, BPS of the respective genes of interest was performed, confirming that *CYP2E1* was significantly hypomethylated in the PD cortex at the CpGs sites investigated (sum score: control 21.58 ± 8.78; PD 11.98 ± 5.3; *p* = 0.001; Figure 2A). These differences were consistently significant also at the level of individual CpG analysis (*p*-values = 0.002–0.005).

In addition, we used a second primer pair for pyrosequencing, encompassing the promoter region of *CYP2E1*. Using these primer pairs, Jiménez-Garza et al. demonstrated the up-regulation of *CYP2E1* methylation in blood from tannery workers exposed to toluene [14]. In the PD cortex, we found significant hypomethylation of *CYP2E1* in all of the CpGs analysed (No. 6–10, Figure 2C,D).

Hypomethylation of *TP73* and *C21ORF56* was also confirmed by pyrosequencing (Figure 2B,C); however, for *CDH13*, only the high methylation level in PD was replicated, whereas the healthy controls seemingly displayed similar levels of methylation at the investigated CpG (PD 93.1 ± 7.75; control 93.4 ± 7.31; *p* = 0.95).

### 3.3. DNA Methylation of CYP2E1 in Skin Fibroblasts and Blood

To determine whether *CYP2E1* methylation might serve as a biomarker in accessible peripheral tissues, we analysed DNA from blood and skin fibroblasts. However, methylation of *CYP2E1* was unchanged when comparing the control and PD in a cohort of 182 vs. 259 samples using primer pairs 1 and 2 (Figure 2B,D). Additional multivariate regression analysis revealed no correlation of *CYPE1* methylation with sex, smoking, or coffee consumption (data not shown).

Blood samples of another independent cohort were grouped according to the reported l-DOPA intake. In addition, to evaluate whether l-DOPA might exert an acute effect on methylation [11], a set of pbms from PD patients never treated with l-DOPA was treated in vitro with l- or d-DOPA.

The methylation of *CYP2E1* DNA was not changed in PD patients treated with different L-DOPA dose ranges in vivo (Pearson correlation coefficient 0.019, *p* = 0.92) nor in pbms treated with l-DOPA in vitro (baseline 22 ± 10%; 100 µM: 24 ± 10%; 200 µM: 22 ± 9%; Figure 2F).

*CYP2E1* was equally methylated in skin fibroblasts derived from PD patients compared with healthy probands (sum of CpG 1–5 (mean ± SD): control: 16 ± 2% vs. PD: 14 ± 3%; *p*-value = 0.8; Figure 2H).

### 3.4. Protein Expression of CYP2E1 in Cortex

Western blot analysis suggested increased expression of CYP2E1 protein in the brains of PD patients compared with those of controls (control: 0.13 ± 0.07 vs. PD: 0.42 ± 0.21; *p* = 0.042; mean ± SD; Figure 2I).

### 3.5. Protein Expression of CYP2E1 in TCE Treated in Cultured Cells

Following treatment with trichloroethylene (TCE), which is known to cause PD in particularly susceptible men, *CYP2E1* protein was up-regulated in a dose-dependent manner (Figure 2J).

### 3.6. SNCA Protein Levels in CYP2E1-Null Mice

A putative influence of *CYP2E1* on SNCA protein levels was tested using *CYP2E1*-null and wild-type (WT) mice. In brain tissue, SNCA protein was significantly decreased in the null mice (*p* = 0.048; ANOVA; Figure 2K).

## 4. Discussion

In this genome-wide methylation analysis, we found 57 differentially methylated CpGs in the cortex of PD patients.

We recently demonstrated hypomethylation of the cytochrome P450 2E1 (CYP2E1) gene and increased expression of CYP2E1 mRNA in the brains of Parkinson’s disease (PD) patients in an independent study using the 27 K micro bead array in a more heterogeneous group of tissues, suggesting that epigenetic variants of this cytochrome (CPY) might contribute to PD susceptibility [5]. Here, we also corroborated the hypomethylation of CYP2E1 at the single-base level using bisulphite-pyrosequencing and demonstrated that the degree of methylation was independent of l-DOPA exposure. CYP2E1 protein was found to be up-regulated in the PD cortex, indicating that the observed hypomethylation is correlated with increased protein expression.

Cytochromes have been implicated in PD pathophysiology, primarily as a putative genetic risk factor [16]. CYP2E1 is of particular interest biochemically and mechanistically because it is located specifically in dopaminergic neurons and plays a key role in the generation of toxic ß-carbolines (1-trichloromethyl-1,2,3,4-tetrahydro-ß-carboline; TaClo) from aldehydes and dopamine metabolites [16,17,18].

Interestingly, the lack of CYP2E1 in the null mice was associated with the down-regulation of alpha-synuclein (SNCA), suggesting that *CYP2E1*, involved in the metabolism of xenobiotics such as trichloroethylene (TCE) and *SNCA* is interconnected and that increased expression of *CYP2E1*, and consequently *SNCA*, might cause neuron damage as a result of exposure to dietary or environmental toxicants that are activated by CYP2E1.

These findings are in line with animal models demonstrating the up-regulation of *SCNA* in dopaminergic SN neurons after TCE exposure that might be mediated by CYP2E1 induction [19].

The observed CYP2E1/SNCA interconnection might be induced by a CYP2E1-dependent increase of oxidative stress, known to cause DNA hypomethylation, including SCNA hypomethylation [20], which was shown previously to result in SCNA protein up-regulation [11].

CYP2E1 is mainly involved in the oxidative metabolism of rather few polar molecules, mainly ethanol and aldehydes [21]. Except for CYP2E1, which is also expressed in dopaminergic neurons of the substantia nigra [16] the brain lacks functionally relevant ethanol and aldehyde-degrading enzyme systems.

CYP2E1 has been implicated in PD pathophysiology at several levels. The induction of CYP2E1 generates reactive oxygen species (ROS) in the substantia nigra of rodents, and CYP2E1-null mice are protected from MPTP toxicity [22,23]. CYP2E affects dopaminergic transmission of the substantia nigra [24]. Transfection of a CYP2E1 expression vector into the macrophage cell lineage sensitized cells to lipopolysaccharide (LPS) stimuli, resulting in increased TNF-alpha expression and activation of p38 signalling [25]. All of these data are in line with our findings of increased CYP2E1 in PD brains.

The hypomethylation of the CYP2E1 gene in the brain allows stronger expression of CYP2E1 in response to a given stimulus, and it was indeed shown that exposure to TCE causes PD in particularly susceptible men [19,26,27] and nigrostriatal degeneration with the development of parkinsonian features in animal models [28,29].

Increased CYP2E1 in humans typically results from ethanol consumption, but trichloroethylene (TCE) is another strong inducer (and preferred substrate) of CYP2E1 [30]. We demonstrated a robust up-regulation of CYP2E1 protein in cultured neuronal cells after TCE exposure.

We did observe hypomethylation of CYP2E1 in peripheral blood in the present study. In addition, in an earlier study of methylation in PD peripheral blood, no change was detected in a cohort of 221 vs. 227 human samples [31]. In another study, allele frequencies—not DNA methylation—of CYP2E1 in DNA derived from the blood of patients with PD and controls were screened, and no significant differences were detected [32]. In contrast, recently, CYP2E1 was found hypermethylated in the peripheral blood of PD patients using a methylation array, but an independent method like pyrosequencing was not applied to confirm these results [33]. Five CpG positions of CYP2E1 analysed by Henderson-Smith were identical to CpGs in our study (Illumina array: cg13315147, cg11445109, cg23400446, cg25530264, cg10862468). One CpG (cg13315147) was hypomethylated in Parkinson’s diseased brains in our survey, but hypermethylated in peripheral blood in the study of Henderson-Smith. The remaining four CpGs were hypomethylated in PD brains (our study) and unchanged from the control in blood [33]. We cannot explain the conflicting results of cg13315147, which might be due to different PD medication in both cohorts, but the other four CpGs also analysed by Henderson-Smith underline our finding of unchanged CYP2E1 methylation status in the peripheral blood of PD patients.

It is uncertain whether the epigenetic dysregulation/hypomethylation of CYP2E1 observed here might represent the epigenetic remnant of an inherited change upon environmental exposure of anteceding generations to solvents [34], rendering the actual carriers of the hypomethylated epigenetic variant of CYP2E1 more vulnerable.

Significant differences were also detected in CpGs related to the MSRA, TP73, and CDH13 genes. MSRA plays a critical role in the antioxidant response, protecting cells from oxidative damage. Cells with increased levels of MSRA are resistant to oxidative stress. It efficiently reduces oxidized alpha-synuclein, which otherwise tends to form potentially neurotoxic protofibrils [35]. The CpGs found to be hypomethylated in the present study are located in the *MSRA* gene body. Intragenic DNA methylation is generally correlated with increased transcriptional activity, and hypomethylation can decrease transcription [36]. Thus, we speculate that the observed intragenic hypomethylation may cause decreased MSRA expression, resulting in reduced antioxidant protection in PD.

Transactivating isoforms of p73 (Tp73) have p53-like pro-apoptotic activities [37]. Hypomethylation of the CpGs located in regulatory elements (TSS1500, CpG island shore) suggests an epigenetic-driven up-regulation of TP73 expression, potentially resulting in neuronal cell death.

Cadherin 13 (*CDH13*) protects vascular endothelial cells from apoptosis due to oxidative stress. While the relevance of our observation to gene expression and its contribution to PD aetiopathogenesis remains unclear, *CDH13* is known to be required for insulin release and thus contributes to the regulation of insulin secretion [38]. Indeed, the onset of diabetes before the onset of PD appears to be a risk factor for more severe PD symptoms.

Furthermore, we found *C21ORF56*, chromosome 21 open reading frame 56, with several hypomethylated CpGs per gene. C21orf56 is an uncharacterized protein, and data regarding its gene function do not exist.

## 5. Conclusions

**Figure 3:**
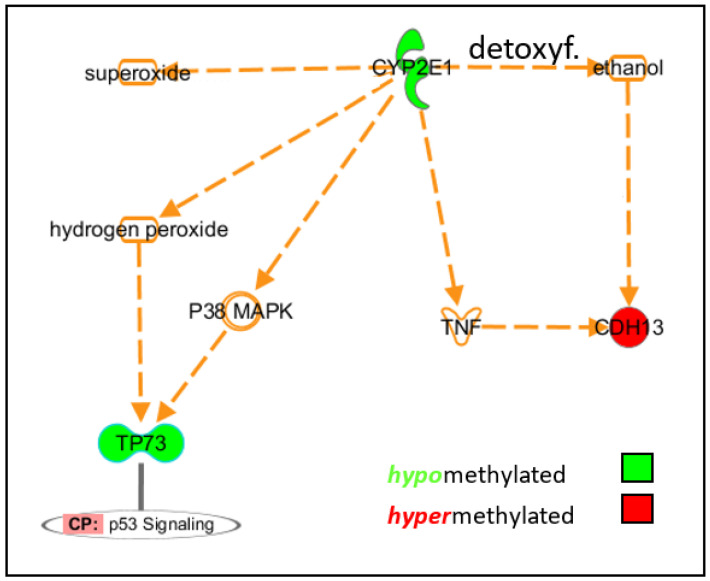
Pathway analysis. IPA Ingenuity pathway analysis showed an interconnected cohort of hypomethylated genes, previously associated with oxidative stress and TNF-alpha up-regulation (CYP2E1) and p53-like pro-apoptotic activities (TP73). Detoxyf. denotes for detoxification; arrows denote increase; minus sign denotes decrease.

The brains of PD patients and healthy individuals revealed different methylation patterns, including those of several candidate genes involved in PD pathology and oxidative stress (Figure 3). In particular, the dysmethylation of *CYP2E1* observed in the brain was not found in peripheral blood or skin fibroblasts, indicating the tissue specificity of CYP2E1 methylation. Furthermore, we found an association of the expression of *SNCA* and *CYP2E1*.

## Figures and Tables

**Table 1:** Differentially methylated genes and CpGs in human cortex comparing PD-affected cases with healthy individuals.

Rank ^1^	Target ID ^2^	Gene ^3^	Chr. ^4^	Control ^4^	PD	Difference ^5^	*p*-Value	Adjusted *p*-Value
**Genes hypomethylated in PD**								
1	cg26077133	MSRA	8	1.375	−0.5731	−1.9481	0.0028	0.6990
2	cg21388339	TP73	1	1.4532	−0.4532	−1.9064	0.0006	0.6547
3	cg00424152	B3GNT7	2	1.9356	0.196	−1.7396	0.0046	0.7104
4	cg13573375	PIAS4	19	1.3363	−0.2018	−1.5381	0.0038	0.7041
5	cg13732083	C21orf56	21	−0.3452	−1.7818	−1.4366	0.0066	0.7352
6	cg25708755	PTPRN2	7	3.8666	2.4326	−1.434	0.0002	0.6339
7	cg05896524	C21orf56	21	0.2687	−1.0967	−1.3654	0.0086	0.7445
8	cg10296238	C21orf56	21	1.6757	0.375	−1.3007	0.0036	0.7028
9	cg02898977	TM9SF1	14	3.5181	2.2475	−1.2706	0.0009	0.6608
10	cg11445109	CYP2E1	10	−1.633	−2.8688	−1.2358	0.0042	0.7086
11	cg04563766	PRKACG	9	1.6484	0.4168	−1.2316	0.0033	0.7028
12	cg23400446	CYP2E1	10	−0.7482	−1.978	−1.2298	0.0059	0.7226
13	cg09672255	ZNF879	5	1.9644	0.7365	−1.2279	0.0056	0.7162
14	cg17763566	HLA-DPB2	6	2.9618	1.7579	−1.2039	0.0013	0.6766
15	cg25545878	C21orf56	21	0.3828	−0.8175	−1.2003	0.0046	0.7105
16	cg19299952	MOBKL2A	19	4.3039	3.1272	−1.1767	0.0081	0.7393
17	cg12016809	C21orf56	21	0.3056	−0.8639	−1.1695	0.0061	0.7288
18	cg08925606	BPIL1	20	2.8527	1.6876	−1.1651	0.0007	0.6608
19	cg07747299	C21orf56	21	−0.0209	−1.1486	−1.1277	0.0043	0.7092
20	cg13315147	CYP2E1	10	−0.5431	−1.6485	−1.1054	0.0034	0.7028
21	cg23026554	LOC441666	10	−2.2328	−3.3233	−1.0905	0.0024	0.6948
22	cg18093448	WWC2	4	1.774	0.705	−1.069	0.0004	0.6339
23	cg20965743	ASPG	14	−0.8006	−1.8428	−1.0422	0.0004	0.6065
24	cg24753094	THSD4	15	2.386	1.3613	−1.0247	0.0038	0.7028
25	cg24530264	CYP2E1	10	−1.3224	−2.3267	−1.0043	0.0096	0.7445
26	cg10862468	CYP2E1	10	−0.8401	−1.7957	−0.9556	0.0025	0.6948
27	cg24136292	INSC	11	1.2508	0.3108	−0.94	0.0022	0.6948
28	cg21787989	LGALS8	1	2.5081	1.6097	−0.8984	0.0037	0.7028
29	cg04398451	MYO15A	17	3.0648	2.1705	−0.8943	0.0095	0.7445
30	cg19039925	GVIN1	11	2.9298	2.1079	−0.8219	0.0072	0.7377
31	cg10309386	BEND7	10	0.9543	0.1425	−0.8118	0.0055	0.7153
32	cg00631877	PLXNC1	12	1.7795	1.0282	−0.7513	0.0015	0.6796
33	cg25094735	NAPSB	19	1.8552	1.1323	−0.7229	0.0028	0.6990
34	cg11133658	SCARA5	8	3.6407	2.9677	−0.673	0.0056	0.7165
35	cg24705404	DAB1	1	0.8954	0.3551	−0.5403	0.0055	0.7157
**Genes hypermethylated in PD**								
1	cg07179329	CDH13	16	−1.0161	3.2007	4.2168	2.41 × 10^−^^6^	0.2671
2	cg21498547	DLGAP2	19	−2.498	0.2503	2.7483	0.0055	0.7157
3	cg00713204	BANP	16	−1.6172	0.2791	1.8963	0.0003	0.6339
4	cg06051619	DIP2C	10	−1.0376	0.8079	1.8455	0.0008	0.6608
5	cg05385718	D2HGDH	2	−0.1851	1.6527	1.8378	0.0092	0.7445
6	cg22508145	CPAMD8	19	0.2203	2.0561	1.8358	0.0055	0.7142
7	cg27649396	C11orf53	11	0.3793	2.1594	1.7801	0.0030	0.7000
8	cg11716267	LOC375190	2	−0.9379	0.6719	1.6098	0.0025	0.6948
9	cg24861399	MGAT5	2	−1.1351	0.3973	1.5324	0.0057	0.7197
10	cg02938066	NFKBIL2	8	1.4743	2.954	1.4797	0.0038	0.7028
11	cg23633026	RASL10B	17	1.7681	3.223	1.4549	0.0018	0.6831
12	cg19680693	GPR83	11	−0.5695	0.8113	1.3808	0.0034	0.7028
13	cg10224537	B3GALT1	2	2.1048	3.4393	1.3345	0.0004	0.6339
14	cg25790212	IGSF9B	11	−1.7987	−0.4692	1.3295	0.0005	0.6489
15	cg09746326	DCUN1D2	13	1.559	2.7568	1.1978	0.0061	0.7288
16	cg18662228	AGAP1	6	−1.0355	0.1227	1.1582	0.0066	0.7352
17	cg12687426	KCNMB3	3	2.2947	3.3274	1.0327	0.0001	0.6065
18	cg04381873	LOC284412	19	2.0913	2.9199	0.8286	0.0036	0.7028
19	cg11342941	AGPAT4	6	2.9545	3.7636	0.8091	0.0054	0.7142
20	cg06979412	RFC3	13	2.9372	3.6803	0.7431	0.0092	0.7445
21	cg22402398	FGR	1	3.0874	3.8231	0.7357	0.0077	0.7377
22	cg08955548	PTPRN2	7	1.3085	1.9535	0.645	0.0019	0.6845

^1^ Rank based on decreasing difference between methylation of control to PD group. No. 1–35 are hypomethylated in PD; No. 1–22 are hypermethylated in PD. ^2^ Target ID based on the annotation of Illumina’s 450 K methylation chip. ^3^ Associated Genes. ^4^ M-value of CpG methylation. ^5^ Difference mean methylation of PD group—mean methylation of control group. Negative sign indicates hypomethylation in PD group. Positive sign indicates hypermethylation of CpG in PD group.

## Data Availability

Details regarding data supporting can be asked from the corresponding author.
